# Foliar Application of TiO_2_ Alleviates the Adverse Effects of Late Sowing by Optimizing Photosynthetic Physiology, Yield, and Quality in Wheat

**DOI:** 10.3390/plants15050840

**Published:** 2026-03-09

**Authors:** Wenqiang Tian, Meilin Hu, Shan Yu, Jun Zhang, Xuehui Wang, Guangzhou Chen, Weijun Yang, Shubing Shi, Jianhua Wang, Jinshan Zhang

**Affiliations:** 1 College of Agronomy, Xinjiang Agricultural University, Urumqi 830052, China; tianwenqiang1206@126.com (W.T.); a0609hml@163.com (M.H.); 19812006521@163.com (X.W.); chenguangzhou668@163.com (G.C.); 1984_ywj@163.com (W.Y.); ssb@xjau.edu.cn (S.S.); 2 Tumushuke Vocational and Technical College, Tumushuke 843900, China; xjausz@126.com; 3 Agricultural Science Research Institute of the Seventh Division of Xinjiang Production and Construction Corps, Kuitun 833200, China; qq1342719948@outlook.com (J.Z.); wangjh63@cau.edu.cn (J.W.); 4 College of Agronomy, China Agricultural University, Beijing 100193, China

**Keywords:** TiO_2_, late-sown wheat, photosynthetic physiology, yield, quality

## Abstract

Late-sown wheat, which misses the optimal photoperiod and temperature for growth, suffers irreversible losses in both grain number per spike and thousand-grain weight, resulting in severe yield reductions. To this end, a two-year field experiment was conducted to evaluate the effects of application timing (S1 at the booting stage and S2 at the flowering stage) and concentration (T0 = 0 μmol L^–1^, T1 = 376 μmol L^–1^, T2 = 501 μmol L^–1^, T3 = 626 μmol L^–1^) on the photosynthetic physiology, grain number per spike, thousand-grain weight, and quality of late-sown wheat, aiming to elucidate the mechanism by which TiO_2_ enhances the yield quality–efficiency relationship in wheat. The results showed that the foliar application of TiO_2_ significantly enhanced the accumulation of photosynthetic pigments (*SPAD*) and spectroscopic indices (*CHI*, *PRI*) in wheat flag leaves, markedly improved the net photosynthetic rate, and increased the activities of antioxidant enzymes (*SOD*, *POD*) while reducing the accumulation of membrane lipid peroxidation products (*MDA*), with the T2 treatment exhibiting the most pronounced effect. Foliar application of TiO_2_ at the S1 stage significantly increased the number of florets and spikelets, improved grain setting rates, and consequently boosted the grain number per spike. Application of TiO_2_ during the S2 stage significantly enhanced grain filling rates, thereby increasing thousand-grain weight and achieving yield improvement. T2 demonstrated optimal performance under both conditions, enhancing grain storage capacity and morphological traits. This approach not only increased late-sown wheat yields but also improved grain quality indicators such as protein content, wet gluten, and sedimentation value. Therefore, applying 501 μmol L^–1^ (T2) TiO_2_ during the booting stage (S1) appears to be effective for achieving high yields and superior quality in late-sown wheat.

## 1. Introduction

Wheat (*Triticum aestivum* L.), as one of the world’s most widely cultivated and highest-yielding staple crops, provides about 20% of the energy and protein in the human diet. It is a vital crop for maintaining global food security and nutritional health [1]. China is the world’s largest producer and consumer of wheat, with stable wheat yields directly related to the nation’s food security strategy and socio-economic stability [2]. As one of the important wheat-producing areas in China, Xinjiang has abundant solar and thermal resources alongside significant diurnal temperature variations. Relying on its oasis and river valley irrigation systems, it has cultivated unique ecological and technological advantages that are conducive to the simultaneous improvement of wheat yield and quality [3]. In recent years, with advances in variety improvement and cultivation techniques, wheat yields in Xinjiang have risen steadily, establishing a model for leveraging regional agricultural strengths and safeguarding national food security [4,5]. However, the region is limited by land resources, degraded arable soil quality, and seasonal water shortages [6,7], leading to widespread late sowing of wheat. This seriously restricts further improvement in wheat yield and quality.

Xinjiang is a typical region characterized by surplus capacity for one crop cycle but insufficient capacity for two. In order to achieve multi-harvest planting and improve land utilization efficiency, the local government has vigorously promoted crop rotation systems such as wheat–maize rotation. However, due to the delayed harvest of the preceding crop, the sowing period for wheat is significantly postponed [8]. Moreover, as the main producing area for high-value crops such as cotton and sunflowers, the region has experienced aggravated soil degradation due to long-term continuous cropping, and the introduction of wheat rotation can improve soil conditions [9,10]. At the same time, late sowing of wheat conserves pre-winter water resources, adapting to the local irrigation system that depends on snowmelt and prevailing autumn water scarcity [11]. Moreover, late sowing offers advantages such as alleviating labor and machinery shortages and achieving a higher yield than spring wheat, leading to a continuous expansion of late-sown wheat cultivation in the region [7,12]. However, late sowing results in insufficient effective accumulated temperature before winter, which is detrimental to tillering. Coupled with inadequate growth, this increases winter mortality, directly limiting the formation of spikelets. Meanwhile, delayed growth results in a mismatch with optimal light and temperature resources during spring, which in turn limits spike grain number and thousand-grain weight, eventually leading to a systematic decline in yield [13].

To overcome the problem of reduced tillering resulting from late sowing, an increase in the seeding rate to raise the number of initial plants and main stems has been widely applied as a cultivation strategy in wheat production. This method directly offsets the shortage of individual tiller spikes and main stem spikes by utilizing the benefit of higher plant population density [7,14,15]. However, the development of both grain number and thousand-grain weight largely relies on proper synchronization with favorable light and temperature conditions. Therefore, the losses arising from ecological mismatch due to late sowing cannot be efficiently corrected through simple agronomic management practices alone [13,16]. Temperature is one of the key factors influencing the formation of spike grain number and thousand-grain weight, with high temperatures exerting a sequential stress effect during their development [17]. Early warming accelerates wheat growth, shortens the vegetative growth period, weakens the material foundation, inhibits spike differentiation, and is detrimental to spikelet formation [18]. High temperature in the middle stage aggravates floret degeneration and directly reduces the number of potential grains per spike [19]. High temperature in the late stage, when accompanied by dry hot wind, seriously hinders grain filling, leading to a decline in thousand-grain weight [20]. Therefore, alleviating the decrease in grain number per spike and thousand-grain weight caused by ecological mismatch through innovative or optimized field management practices has become a core issue requiring urgent resolution to break through the yield bottleneck in late-sown wheat and to achieve high and stable yields.

Titanium ranks ninth in abundance within the Earth’s crust, with a content about 100 times that of copper, accounting for roughly 0.57% of the crust’s total weight. Its most significant compound, TiO_2_, as an emerging type of plant regulator, demonstrates potential value in modulating crop photosynthesis, stress resistance, and production quality, offering a possible physiological regulatory pathway for achieving high yields and superior quality in crops under adverse conditions [21,22,23,24]. At present, it has been applied in flax [25], broad bean [26], tomato [23], peanut [27], sunflower [28], and other crops. It has also been confirmed that foliar spraying of titanium significantly enhances antioxidant enzyme activity and photosynthetic pigment content, particularly chlorophyll in crops. This effectively alleviates stress conditions, synergistically improves crop photosynthetic production capacity, and provides a guarantee for improving crop yield and quality. In addition, precise intervention during the critical period of yield component formation is necessary to achieve efficient coupling between external regulation and internal physiological processes, thereby fully realizing the potential of titanium for enhancing yield and stress resistance [29]. Existing research indicates that within the recommended dosage range, spraying titanium fertilizer on the leaves has not been reported to cause phytotoxicity to crop growth [28,30], nor have any adverse effects on human health been identified in agricultural products treated with foliar titanium sprays [31]. Therefore, determining the optimal application timing and concentration of titanium preparations, and systematically integrating these with high-application-rate field management practices to form a precise spraying technology scheme capable of effectively compensating for yield-limiting factors (spikelet number, grain number, and grain weight) caused by late sowing, has become an urgent technical challenge to be resolved for achieving high and stable yields in late-sown wheat.

Therefore, the purpose of this experiment was to determine the optimal spraying period and dosage of TiO_2_ to enhance both the grain number per spike and the thousand-grain weight in late-sown wheat, thereby increasing wheat yield. It further seeks to elucidate the mechanism by which photosynthetic physiology is regulated to achieve yield increases and quality improvements. Through two years of field experiments, TiO_2_ was sprayed at varying concentrations during different growth stages, and its comprehensive effects on photosynthetic physiology, resistance enzyme activity, spikelet number composition, grain filling, and yield quality of late-sown wheat were systematically evaluated. This study provides valuable insights into enhancing wheat yield and production efficiency.

## 2. Materials and Methods

### 2.1. Experimental Site

**Figure 1:**
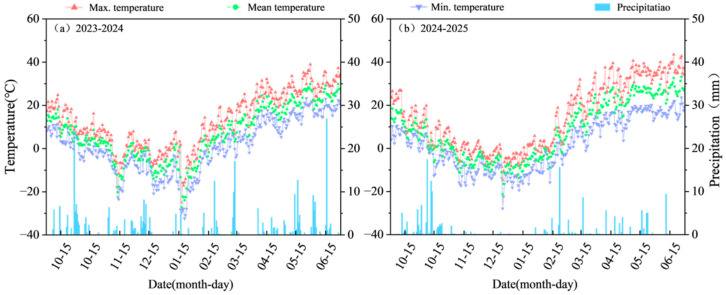
Daily temperature and daily rainfall during the wheat growing season in the experimental site from 2023 to 2025.

The field experiment was conducted from September 2023 to July 2025 at the Institute of Agricultural Sciences, Tacheng Prefecture (46°21′ N, 82°41′ E; altitude 415 m). This region is a typical area for late-sown wheat cultivation. According to the Köppen climatic classification, it is classified as BSk (semi-arid steppe), with an annual average temperature of 6.2 °C, an annual total number of sunshine hours of 2832 h, and average annual precipitation of 270 mm. The daily temperature and precipitation during the wheat growing season are shown in Figure 1. The previous crop in the two-year experiment was corn, and the soil type was loam. The physical and chemical properties of the surface soil are shown in Table 1.

### 2.2. Experimental Design and Field Management

The test wheat variety was Xindong 18, which is prevalently planted in the local area. The test reagent was organic chelated titanium 12% raw powder, a white solid powder readily soluble (ordinary small particle form, not nanomaterial) in water with an aqueous solution pH of 6.0. The main components comprised 12% titanium dioxide (TiO_2_), 33% organic chelating substance, and 12% amino nitrogen (manufactured by Yangling Aobang Biological Science Co., Ltd., Xianyang, China).

The field experiment employed a split-plot design, with the main plots being the period of foliar spraying of TiO_2_, comprising the booting stage (S1) and flowering stage (S2). The spraying concentration of TiO_2_ was set as the sub-plot, with 0 μmol L^–1^ (T0), 376 μmol L^–1^ (T1), 501 μmol L^–1^ (T2), and 626 μmol L^–1^ (T3), totaling eight treatments. The plot area was 10.0 m^2^ (2.0 m long and 5.0 m wide), with four replicates.

The sowing dates in the two years were 15 October 2023 and 19 October 2024, respectively, with a seeding rate of 360 kg ha^–1^ (this optimal rate for late-sown wheat was determined in prior research by this group). Artificial strip sowing was conducted with a row spacing of 20 cm and a sowing depth of 4 cm. No drip irrigation was provided for seedling water and overwintering water before winter. Drip irrigation was applied using laid drip tape after spring, with one tube serving three rows and spaced 60 cm apart. All other field management practices followed local high-yield production standards and remained uniform across all treatments.

### 2.3. Measurements

#### 2.3.1. *SPAD* Value

*SPAD* values were measured using a chlorophyll meter (SPAD-502, Konica Minolta, Inc., Tokyo, Japan) at 0, 5, 10, 15, 20, 25, and 30 days after the application of TiO_2_. Five plants showing uniform growth were selected from each plot for fixed-point measurements, and each leaf was measured from the base to the tip, with the mean value recorded. The mean value of the five plants was calculated to represent the *SPAD* value of each plot.

#### 2.3.2. Reflective Spectral Parameters

During the 2024–2025 wheat growing season, a handheld spectroradiometer (UniSpec-SC, Hansatech, King’s Lynn, UK) was used to measure the reflectance spectra of wheat flag leaves at different growth stages after TiO_2_ application. Five plants from each plot were evaluated. After processing the original spectral data, reflectance values and reference reflectance data at various wavelengths were obtained. The measurements of the five plants were averaged to obtain a single value per plot for each spectral parameter. The spectral indices were calculated according to the method described in Reference [32], including the modified simple ratio index and photochemical reflectance index using the balance equation, as follows:

(1) *CHI* = (R750 − R445)/(R705 − R445)

(2) *PRI* = (R531 − R570)/(R531 + R570)

where the *CHI* exhibits a positive correlation with chlorophyll content, and the *PRI* serves as an effective indicator reflecting the xanthophyll cycle.

#### 2.3.3. Photosynthetic Parameters

Diurnal variations in photosynthetic parameters were measured using a photosynthetic apparatus (CI-304, CID, Inc., Camas, WA, USA) at the flowering stage and grain filling stage on clear, windless days. Four wheat plants per plot were selected to measure net photosynthetic rate (*Pn*, µmol CO_2_ m^–2^ s^–1^), transpiration rate (*Tr*, mmol H_2_O m^–2^ s^–1^), stomatal conductance (*Gs*, mmol H_2_O m^–2^ s^–1^), and intercellular CO_2_ concentration (*Ci*, µmol CO_2_ mol^–1^). Daily variations were recorded during three time slots: 08:00–10:00, 13:00–15:00, and 18:00–20:00. For each time slot, the measurements from four plants were averaged to obtain plot-level photosynthetic parameters.

#### 2.3.4. Enzyme Contents

In the 2024–2025 wheat growing season, the main spikes of wheat with uniform growth and synchronous flowering were selected for marking in each treatment. Subsequently, flag leaves of ten labeled wheat plants were sampled. Enzyme contents were determined using a kit to measure superoxide dismutase (*SOD*, g mL^–1^), peroxidase (*POD*, g mL^–1^), malondialdehyde (*MDA*, g mL^–1^), glutamine synthetase (*GS*, g mL^–1^), nitrate reductase (*NR*, g mL^–1^), and nitrite reductase (*NIR*, μmol mL^–1^). The assay kits were manufactured by Shanghai Enzyme-Linked Biotechnology Co., Ltd. (Shanghai, China). The determination method was a double-antibody sandwich, with absorbance measured at 450 nm wavelength by a microplate reader. Flag leaf samples from ten labeled plants were pooled and analyzed, and the resulting value was used as the plot-level enzyme content.

#### 2.3.5. Grain Filling Parameters

At the flowering stage, 120 main stem spikes with consistent flowering times were selected from each plot and marked. Following flowering, sampling was performed every five days, and 10 marked main stem spikes were taken each time. Electron microscopy (SZM7045, Shunyu Optical Technology Co., Ltd., Ningbo, China) was used to photograph and observe grains from the middle section of five main-stem spikes. Additionally, ten grains of each spike from the middle section of five other spikes were selected, baked in an oven for 30 min at 105 °C to inactivate enzymes. Subsequently, the samples were dried at 85 °C until a constant weight was achieved; these were weighed and converted to thousand-grain weight for grain filling analysis. At the maturity stage, the physical characteristics of grain length, width, circumference, diameter, area, and roundness were measured for 20 grains collected from the middle portion of the main stem using a wheat grain trait measuring instrument (SC-G, Hangzhou Wanshen Detection Technology Co., Ltd., Hangzhou, China). For grain filling analysis, the thousand-grain weight converted from ten spikes was taken as the plot-level value. For grain morphology, the measurements obtained from 20 grains were averaged to represent each plot.

#### 2.3.6. Yield and Quality

On 15 July 2024 and 2025, 30 wheat spikes were randomly selected from each plot to determine the number of florets, spikelets, and grains per spike, and to calculate the floret and spikelet fertility rates. Representative rows (excluding edge rows) were then selected to determine the number of spikes per plot. These were harvested, manually threshed, and analyzed to determine the thousand-grain weight, from which the yield was derived. Yield was calculated based on the harvested area of each plot and converted to kg hm^–2^. Grain quality parameters were measured using grain samples collected from each plot. A near-infrared grain analyzer (IM9500, Perten Instruments, Hägersten, Sweden) was used to determine grain protein content (*GPC*, %), wet gluten content (*WGC*, %), grain starch content (*GSC*, %), sedimentation value (*SV*, mL), and water absorption (*WA*, %).

### 2.4. Statistical Analysis

All statistical analyses were performed using plot-level means, with each plot serving as the experimental unit. Within each plot, measurements from multiple sampled plants (e.g., five plants for *SPAD* and spectral parameters, ten plants for enzyme contents, five spikes for grain filling analysis, etc.) were averaged (individual plants were not treated as independent replicates) to obtain a single representative value per plot before statistical analysis. The experimental data from the two growing seasons were processed using Microsoft Excel 2021 (Microsoft Corp., Redmond, WA, USA) and analyzed using analysis of variance in SPSS 29.0 software (IBM Corp., Chicago, IL, USA). When a significant treatment effect was observed at *p* < 0.05, Duncan’s multiple range test (Duncan’s) was used to determine differences between treatments. Origin 2019b software (OriginLab, Northampton, MA, USA) was used to generate the figures.

## 3. Results

### 3.1. SPAD Value

**Figure 2:**
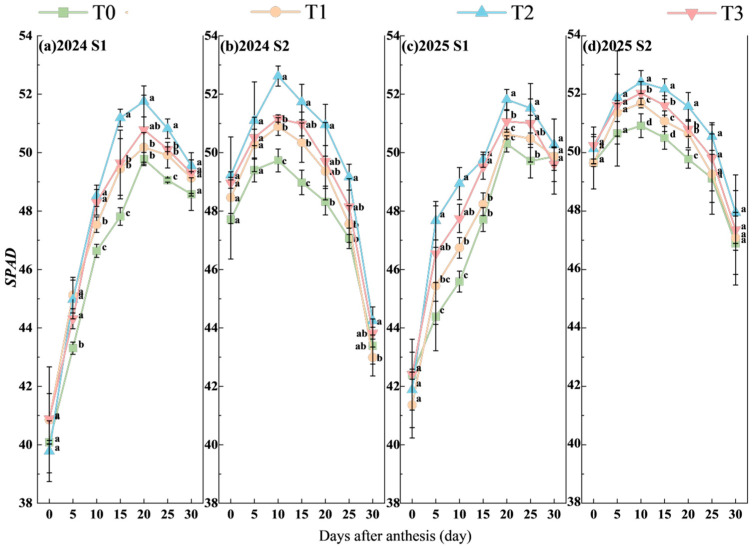
Effects of TiO_2_ spraying time and concentration on *SPAD* value of flag leaf after anthesis in late-sown wheat (days after anthesis). Note: Treatments with a different letter within a year were significantly different based on Duncan’s multiple range test at *p* < 0.05, with vertical bars representing standard errors of the mean. S1 and S2 denote TiO_2_ application during the booting stage (S1) and flowering stage (S2), respectively. T0, T1, T2, and T3 indicate the spraying concentrations of TiO_2_ of 0 μmol L^–1^ (T0), 376 μmol L^–1^ (T1), 501 μmol L^–1^ (T2), and 626 μmol L^–1^ (T3), respectively.

The *SPAD* values of wheat flag leaves after anthesis in the two growing seasons showed an increasing trend with growth progression, reaching a peak at 10 days after flowering before declining. The highest values were observed at 10 days after flowering in both cases (Figure 2). Spraying TiO_2_ significantly increased the *SPAD* values of wheat flag leaves, with consistent results observed over two years. Under S1 and S2 treatments, the *SPAD* values of wheat flag leaves after flowering were highest at T2. Under S1, the two-year peak values at T1, T2, and T3 were significantly increased by 0.73%, 3.49%, and 1.77% on average, respectively, compared with T0. Under S2, the two-year peak values of T1, T2 and T3 were significantly increased by 2.17%, 4.26% and 2.88% on average compared to T0. Furthermore, no significant difference was observed between S1 and S2.

### 3.2. Reflectance Spectral Parameters

**Figure 3:**
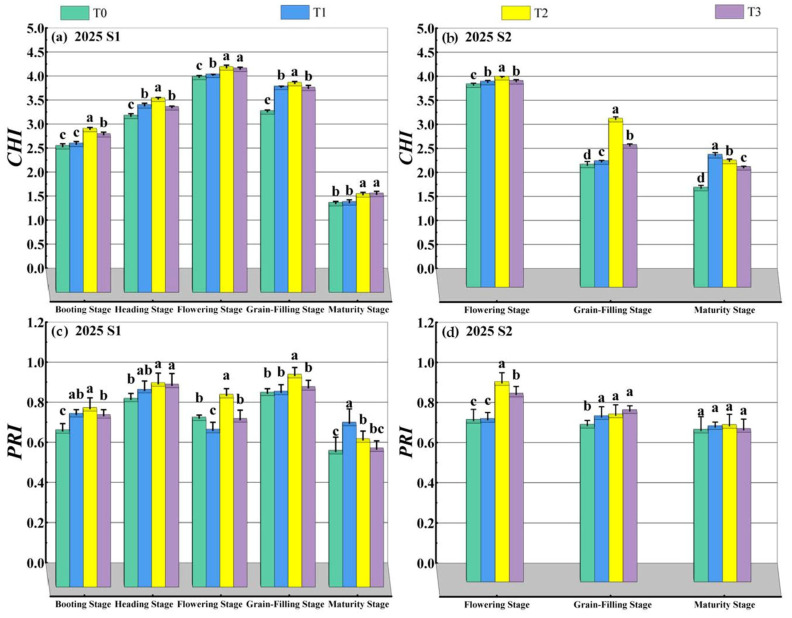
Effects of spraying time and concentration of TiO_2_ on reflectance spectral parameters of late-sown wheat. Note: The *CHI* value is positively correlated with chlorophyll content, whilst the *PRI* value serves as an effective indicator of the xanthophyll cycle. Treatments with different letters within a year were significantly different based on Duncan’s multiple range test at *p* < 0.05, with vertical bars representing standard errors of the mean. S1 and S2 denote TiO_2_ application during the booting stage (S1) and flowering stage (S2), respectively. T0, T1, T2 and T3 indicate the spraying concentrations of TiO_2_ of 0 μmol L^–1^ (T0), 376 μmol L^–1^ (T1), 501 μmol L^–1^ (T2), and 626 μmol L^–1^ (T3), respectively.

As shown in Figure 3, under TiO_2_ foliar application, the *CHI* and *PRI* values of wheat flag leaves significantly increased in both growing seasons and reached peak values at the flowering stage. Under the treatments of S1 and S2, T2 yielded the most favorable results, with no significant difference observed between S1 and S2. The two-year mean CHI values at the flowering stage for T1, T2, and T3 were significantly increased by 1.18%, 4.14%, and 2.83%, respectively, compared to T0, while the *PRI* mean values were significantly increased by 3.50%, 18.64%, and 7.61%, respectively. This indicates that spraying TiO_2_ enhances photosynthetic pigment-related indices and increases the relative pigment content in late-sown wheat flag leaves, with the T2 concentration achieving the most favorable results.

### 3.3. Photosynthetic Parameters

**Figure 4:**
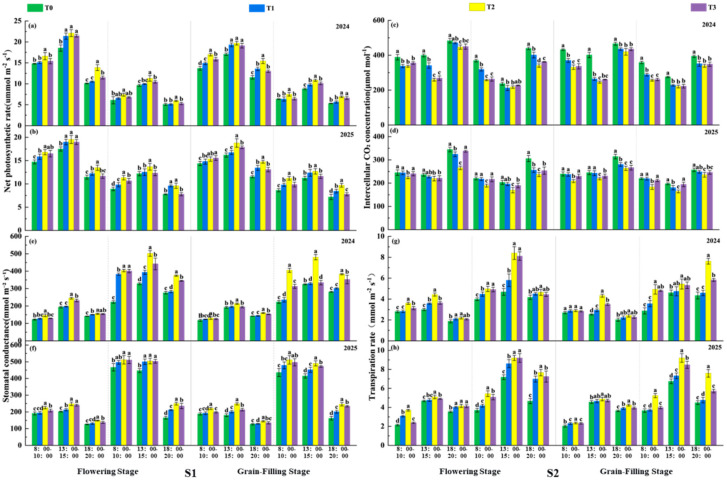
Effect of TiO_2_ application timing and concentration on diurnal variations in photosynthetic parameters during flowering and grain filling stages of late-sown wheat. Note: *Pn*, *Tr*, *Gs* and *Ci* denote net photosynthetic rate, transpiration rate, stomatal conductance and intercellular CO_2_ concentration, respectively. S1 and S2 denote TiO_2_ application during the booting stage (S1) and flowering stage (S2), respectively. T0, T1, T2 and T3 indicate the spraying concentrations of TiO_2_ of 0 μmol L^–1^ (T0), 376 μmol L^–1^ (T1), 501 μmol L^–1^ (T2), and 626 μmol L^–1^ (T3), respectively. (**a**,**b**) reflect the net photosynthetic rate indices, (**c**,**d**) reflect the intercellular carbon dioxide concentration indices, (**e**,**f**) reflect the stomatal conductance indices, and (**g**,**h**) reflect the transpiration rate indices. Different letters indicate significant differences at the *p* < 0.05 level.

The diurnal variation curves of *Pn*, *Gs*, and *Tr* in wheat flag leaves during the flowering and grain filling stages under various treatments exhibited an initial rise followed by a decline, whereas *Ci* showed an inverse trend relative to the other parameters (Figure 4). Data from both growing seasons indicated that spraying TiO_2_ significantly increased *Pn*, *Gs*, and *Tr* of wheat flag leaves while reducing *Ci*. Under the identical spraying concentration, the S1 treatment exhibited average increases of 1.74%, 6.44%, and 7.66% in *Pn*, *Gs*, and *Tr*, respectively, compared to S2 across two years, two periods, and three time slots for flag leaves. During the same spraying period, wheat flag leaves under T2 treatment consistently demonstrated the highest *Pn*, *Gs*, and *Tr* values alongside the lowest *Ci*. Under treatments of T1, T2, and T3, the average *Pn* values of the flag leaf across two years, two periods, and three time slots increased by 8.92%, 19.63%, and 10.04%, respectively, compared to the control (T0). This indicates that spraying TiO_2_ effectively enhances the photosynthetic capacity of late-sown wheat flag leaves, with the S1T2 combination showing the most pronounced effect.

### 3.4. Enzyme Content

**Figure 5:**
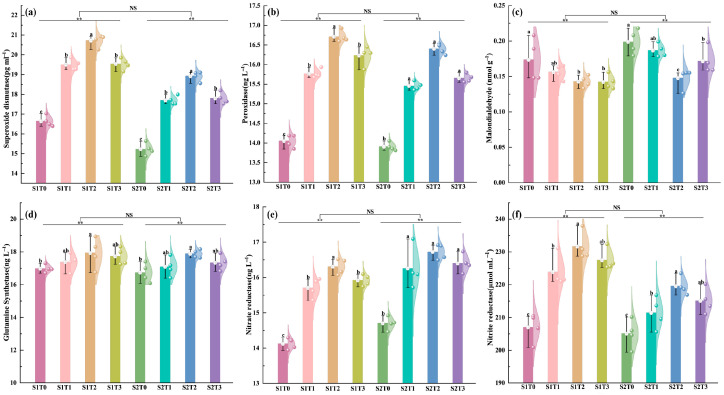
Effects of TiO_2_ spraying time and concentration on enzyme content in flag leaves of late-sown wheat (2024–2025). Note: *SOD*, *POD*, *MDA*, *GS*, *NR* and *NIR* represent the contents of superoxide dismutase, peroxidase, malondialdehyde, glutamine synthetase, nitrate reductase and nitrite reductase, respectively. S1 and S2 denote TiO_2_ application during the booting stage (S1) and flowering stage (S2), respectively. T0, T1, T2 and T3 indicate the spraying concentrations of TiO_2_ of 0 μmol L^–1^ (T0), 376 μmol L^–1^ (T1), 501 μmol L^–1^ (T2), and 626 μmol L^–1^ (T3), respectively. Different letters and "**" indicate significant differences at the *p* < 0.05; *p* < 0.05 level. (**a**) shows the superoxide dismutase content, (**b**) shows the peroxidase content, (**c**) shows the malondialdehyde content, (**d**) shows the glutamine synthetase content, (**e**) shows the nitrate reductase content, and (**f**) shows the nitrite reductase content.

It can be observed from Figure 5 that, compared with the T0 control, TiO_2_ application significantly influenced enzyme contents in wheat flag leaves under all treatments. Specifically, under T1, T2, and T3 treatments, the contents of *SOD*, *POD*, *GS*, *NR*, and *NIR* were significantly increased by 16.74–24.43%, 11.69–18.46%, 2.22–6.22%, 10.90–14.60%, and 5.63–9.50%, respectively, compared with T0. In contrast, *MDA* content was significantly reduced by 7.42–21.99% under TiO_2_ treatments. At the same spraying concentration, the contents of *SOD*, *POD*, *GS*, *NR*, and *NIR* in wheat flag leaves were higher under S1 than under S2, with the exception of *MDA*, although the differences were not statistically significant. At the same spraying period, the contents of key enzymes in flag leaves were highest under the T2 treatment. This indicates that spraying TiO_2_ enhances the antioxidant capacity of flag leaves in late-sown wheat, promotes nitrogen metabolism, reduces the accumulation of membrane lipid peroxidation products, and thereby mitigates the adverse effects of late-sowing stress, with the T2 concentration proving optimal.

### 3.5. Spike Composition

**Figure 6:**
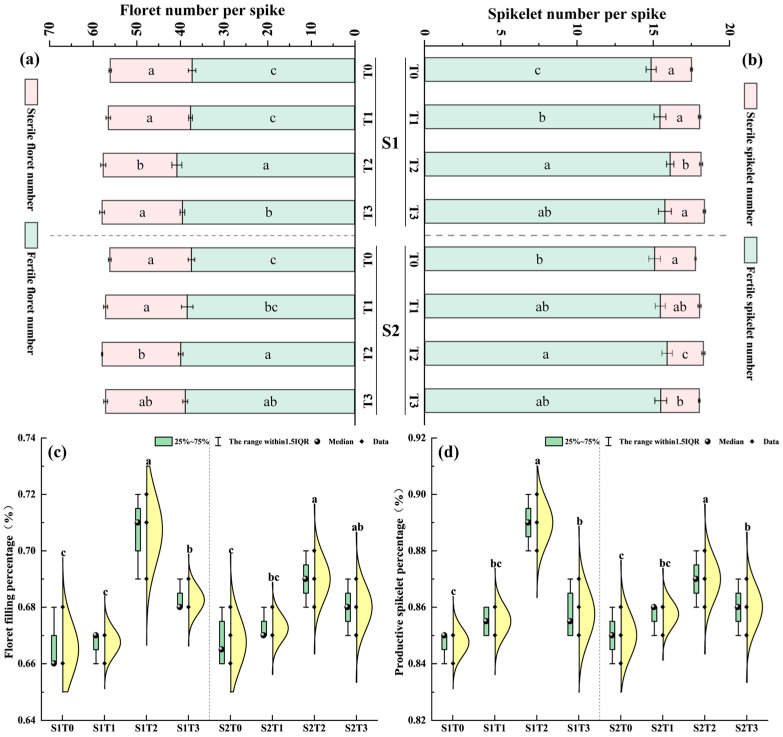
Effects of TiO_2_ spraying period and concentration on the number of florets and spikelets of late-sown wheat (2024–2025). Note: S1 and S2 denote TiO_2_ application during the booting stage (S1) and flowering stage (S2), respectively. T0, T1, T2 and T3 indicate the spraying concentrations of TiO_2_ of 0 μmol L^–1^ (T0), 376 μmol L^–1^ (T1), 501 μmol L^–1^ (T2), and 626 μmol L^–1^ (T3), respectively. Different letters indicate significant differences at the *p* < 0.05 level. (**a**) shows the number of florets per spike, (**b**) shows the number of spikelets per spike, (**c**) shows the floret fertility rate per spike, and (**d**) shows the spikelet fertility rate per spike.

As shown in Figure 6, compared with T0, spraying TiO_2_ significantly increased the number of florets per wheat spike (1.77~8.00%) and spikelets (3.17~6.93%), reduced the number of sterile florets (0.20~7.44%) and sterile spikelets (2.90~21.02%), and increased the floret-to-spikelet conversion rate (0.56~4.89%) and spikelet-to-grain conversion rate (0.88~3.56%). At the same spray concentration, S1 exhibited significantly higher numbers of florets and spikelets than S2, and effective floret and spikelet numbers increased significantly by 0.39% and 0.36%, respectively. At the same application period, the T2 treatment yielded the highest composition of florets and spikelets within the wheat spike. This indicates that spraying TiO_2_ increases the number of effective florets and effective spikelets, reduces the abortion rate, and consequently enhances the potential grain number per spike in late-sown wheat. The combined effect was most prominent when T2 concentration was applied during the S1 stage.

### 3.6. Grain Formation

**Figure 7:**
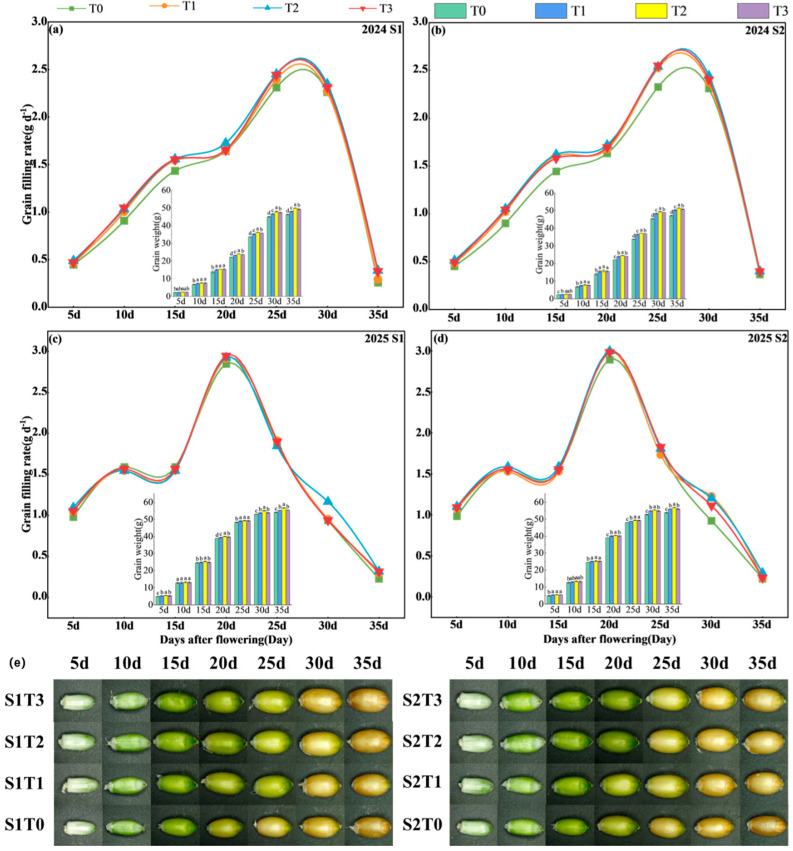
Effects of TiO_2_ spraying time and concentration on grain formation of late-sown wheat. In the figure (**e**), the grain morphology photos were taken every 5 days after flowering. Note: S1 and S2 denote TiO_2_ application during the booting stage (S1) and flowering stage (S2), respectively. T0, T1, T2 and T3 indicate the spraying concentrations of TiO_2_ of 0 μmol L^–1^ (T0), 376 μmol L^–1^ (T1), 501 μmol L^–1^ (T2), and 626 μmol L^–1^ (T3), respectively. (**a**–**d**) shows the wheat grain filling process, and (**e**) shows the changes in wheat grains during filling. Different letters indicate significant differences at the *p* < 0.05 level.

As presented in Figure 7, the grain weight of wheat showed a continuous increasing trend, whereas the grain filling rate generally followed a pattern of initial increase and subsequent decline. However, the timing of the maximum peak between the two growing seasons was not consistent. The application of TiO_2_ significantly increased wheat grain weight and filling rate, while also promoting an increase in grain volume. At the same spraying concentration, no significant difference in grain filling rate was observed between S_1_ and S_2_ treatments, although the two-year average maximum grain weight (35 days after anthesis) of S_1_ increased significantly by 1.80% compared with S_2_. In comparison with T_0_, the two-year maximum average grain weight under T_1_, T_2_, and T_3_ increased by 4.12%, 6.72%, and 5.03%, respectively. The maximum grain filling rate occurred at 25 days after flowering in 2024 and at 20 days after flowering in 2025. Compared with T_0_, the filling rates under treatments T_1_, T_2_, and T_3_ increased by 6.38%, 8.91%, and 7.68%, respectively, during this period in 2024, and by 2.09%, 3.26%, and 2.41% in 2025. Under different spraying periods, the T_2_ treatment resulted in the highest grain weight, fastest grain filling rate, and largest grain volume. These findings indicate that the application of TiO_2_ can promote the development of late-sown wheat grains, increase grain weight and grain filling rate, with T_2_ concentration being the optimal application rate. However, the spraying timing had a relatively minor effect on the grain filling process, and the spraying period of S1 showed the best performance on grain weight.

### 3.7. Grain Morphology

**Figure 8:**
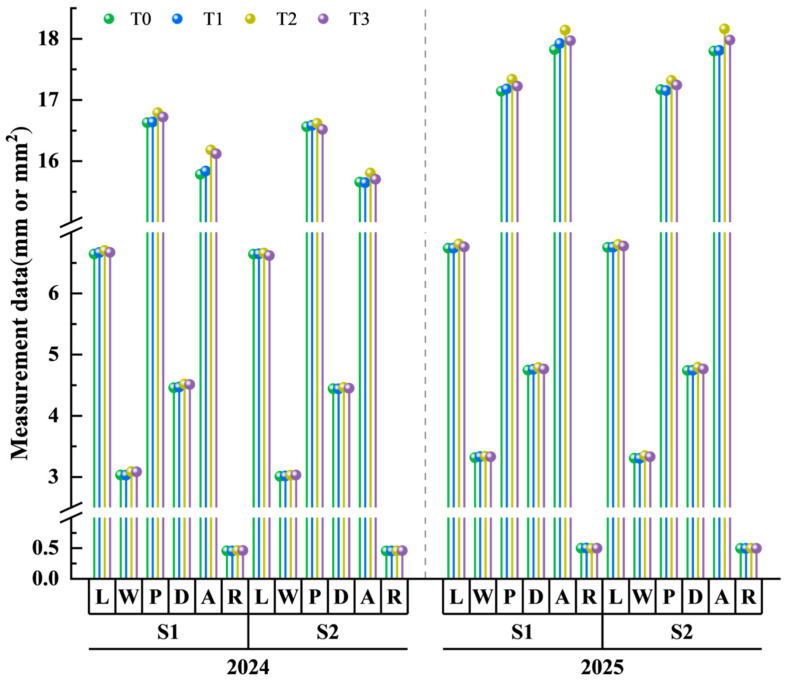
Effects of TiO_2_ spraying time and concentration on grain morphology of late-sown wheat. Note: In the figure, L, W, P, D, A and R represent grain length, width, perimeter, diameter, area and roundness, respectively. S1 and S2 denote TiO_2_ application during the booting stage (S1) and flowering stage (S2), respectively. T0, T1, T2 and T3 indicate the spraying concentrations of TiO_2_ of 0 μmol L^–1^ (T0), 376 μmol L^–1^ (T1), 501 μmol L^–1^ (T2), and 626 μmol L^–1^ (T3), respectively.

As illustrated in Figure 8, under TiO_2_ foliar application treatment, the length, width, perimeter, diameter, and area of wheat grains increased during both growing seasons, with only slight changes in roundness. In addition, grain morphological indices showed better performance in the 2024–2025 growing season. Compared with T0, the grain area under treatments T1, T2, and T3_3_ increased significantly by 0.13%, 1.73%, and 1.20% during the 2023–2024 season, and by 0.31%, 1.91%, and 0.91% during the following season. At the same application concentration, all grain morphological indices of S1 significantly outperformed S2. At different application periods, the T2 treatment demonstrated the most pronounced improvement in various morphological characteristics of grains. This indicates that TiO_2_ application effectively enhances grain storage capacity and plumpness in late-sown wheat, ensuring grain filling. The S1T2 combination effect was the most prominent and exhibited consistent performance across years.

### 3.8. Yield and Yield Components

The results from the two growing seasons in Table 2 indicated that foliar application of TiO_2_ consistently increased wheat yield. S1 achieved this increase by boosting grain number per spike, whereas S2 increased yield by enhancing thousand-grain weight. However, neither S1 nor S2 significantly affected the number of spikes. Compared with T0, the average value over the two-year period of grain number per spike increased by 3.67%, 8.87%, and 4.85% under T1, T2, and T3 treatments, thousand-grain weight increased significantly by 2.36%, 5.73%, and 3.02%, and yield increased significantly by 1.63%, 5.05%, and 3.39%, respectively. At the same spraying concentration, the two-year average grain number per spike, thousand-grain weight, and yield under S1 treatment significantly increased by 5.96%, 1.97%, and 1.73%, respectively, compared to S2. At different application stages, the T2 treatment yielded the most favorable effects on both yield and yield components. This indicates that spraying TiO_2_ can enhance the yield of late-sown wheat. Overall, spraying at T2 concentration during the S1 stage achieved the most significant increase in yield, which appears to be an effective strategy for achieving stable and increased production of late-sown wheat.

### 3.9. Grain Quality

As presented in Table 3, the application of TiO_2_ decreased starch content in late-sown wheat while increasing protein content, wet gluten content, water absorption rate, and sedimentation value, thereby enhancing wheat quality during the two growing seasons. Under the same spraying concentration, the average grain protein content in S_1_ increased significantly by 1.14% compared with S_2_ over the two years, whereas the average starch content decreased significantly by 0.13%. In comparison with T_0_, the mean starch content of grains in T_1_, T_2_, and T_3_ decreased significantly by 0.12%, 0.52%, and 0.32%, respectively; the mean protein content increased significantly by 1.27%, 3.4%, and 2.62%; the mean wet gluten content increased by 1.27%, 3.84%, and 2.74%; the mean water absorption capacity increased by 0.69%, 1.37%, and 0.99%; and the mean sedimentation value increased by 1.56%, 5.74%, and 3.67%, respectively. Under S1 and S2 treatments, the T2 treatment demonstrated the most obvious effect on improving wheat quality. This indicates that spraying TiO_2_ enhances the processing and nutritional quality of late-sown wheat, with the S1T2 treatment providing the most favorable quality improvement. This provides a feasible strategy for simultaneously achieving high yield and superior quality.

## 4. Discussion

### 4.1. Optimizing the Photosynthetic Physiology of Late-Sown Wheat Through Enhanced Photosynthetic Parameters and Stress-Resistant Enzyme Content via TiO_2_

Flag leaves, which function as the main photosynthetic assimilation organs in wheat, exert a direct effect on photosynthetic capacity through their physiological condition. This subsequently regulates the transport of photosynthetic products to the grain, thereby influencing yield establishment [33]. Previous studies have shown that the application of TiO_2_ increases the core pigment content in leaves of crops such as chickpea [34], lentil [35], eggplant [36], and spinach [37]. This supports photoreactions while providing a more suitable basis for carbon dioxide fixation during dark reactions, thereby improving overall photosynthetic capacity. However, when the concentration of TiO_2_ applied is excessively high, the leaf pigment content is reduced, resulting in the inhibition of photosynthesis [38]. In this study, it was confirmed that spraying TiO_2_ enhances the flag leaf pigment response index, including *SPAD*, *CHI*, and *PRI* values, thereby elevating the net photosynthetic rate of wheat. With increasing concentration, the promoting effect gradually decreases, which is consistent with previous research findings. Spraying titanium on maize leaves can strengthen the xanthophyll cycle in leaves; mitigate photo-oxidative damage, consequently prolonging the functional period of leaves; and improve photosynthetic efficiency [39]. This demonstrates that foliar application of TiO_2_ can mitigate the adverse effects of accelerated growth, premature leaf senescence, and reduced photosynthetic duration caused by high temperature stress in late-sown wheat to some degree [7]. Furthermore, foliar application of titanium can enhance photosynthesis, increase the content of stress-related enzymes, activate antioxidant defenses, and alleviate oxidative stress [40]. Spraying TiO_2_ can increase the content of proline and malondialdehyde alongside antioxidant activity, stabilize chloroplast structure and function, and mitigate stress damage as well [41]. The findings of this study indicated that foliar application of TiO_2_ to late-sown wheat enhances the content of antioxidant enzymes (*SOD*, *POD*), reduces the accumulation of oxidative damage products (*MDA*), and increases the content of key nitrogen assimilation enzymes (*GS*, *NR*, and *NIR*) in the flag leaf, improving the tolerance of late-sown wheat to resist high temperature stress while simultaneously promoting the transformation of inorganic nitrogen, thereby maintaining high photosynthetic productivity under adverse conditions. This experiment also showed that foliar application at concentration T2 yielded the most favorable promotional effect, whereas increasing the concentration to T3 resulted in weakened stress resistance. Nevertheless, the spraying time showed no significant influence on the contents of major photosynthetic pigments and the activities of antioxidant enzymes in flag leaves, whereas application at the booting stage (S_1_) was found to be more effective in regulating photosynthetic parameters such as net photosynthetic rate (P_n_). This effect may be explained by the earlier application that enabled the initiation of photosynthetic optimization at an earlier stage, thereby producing a cumulative impact over a longer duration, which is reflected in improved photosynthetic performance. Consequently, more time is made available for the accumulation of photosynthetic assimilates and their translocation to grains, resulting in more efficient conversion into yield formation advantages. In contrast, the contents of key photosynthetic pigments and antioxidant enzyme activities remained relatively unaffected by spraying time, which may be due to their synthesis and metabolic regulation being more directly governed by TiO_2_ concentration rather than the timing of application.

### 4.2. Enhancing Late-Sown Wheat Yields by Synergistically Increasing Both the Number of Grains per Spike and the Thousand-Grain Weight via TiO_2_

Due to the delayed growth period, late-sown winter wheat is susceptible to high-temperature stress during the later stages of development, which is not conducive to the formation of grain number per spike and thousand-grain weight, ultimately resulting in reduced yields [13,16]. The formation of grain number per spike is closely related to the number of florets and spikelets produced during the differentiation of young spikes at the booting stage and their seed setting rate [42,43]. It was observed in this study that the application of TiO_2_ at the heading stage increased the differentiation of florets and spikelets in late-sown wheat, enhanced the seed setting rate, reduced the number of abortions, and led to an increase in the number of grains per spike. The development of thousand-grain weight is limited by the grain filling rate and is associated with grain volume [7,44]. It was further found that the application of TiO_2_ at the flowering stage increased the grain filling rate of wheat while promoting grain morphogenesis (such as increased grain length, width, and area), thereby resulting in higher thousand-grain weight. Previous studies have shown that foliar application of titanium improves crop yield, although the mechanisms responsible for yield improvement differ depending on the application timing [29,34,35,38]. The increase in yield under S_1_ treatment was mainly achieved through the enhancement in the number of grains per spike. The physiological basis may be that earlier application of titanium fertilizer enables better optimization of photosynthetic performance and stress tolerance in plants, thereby ensuring grain formation per spike. Concurrently, it exerted a certain degree of promotion on the enhancement of thousand-grain weight. The spraying period of S2 was later, with a shorter duration of action, yet its effects were concentrated, enabling more direct intervention during the grain filling stage. This effectively promoted increased grain weight and achieved yield enhancement. Compared to S2, S1 demonstrated a more pronounced yield-enhancing effect. This was due to the extended period of action under S1, which encompasses multiple key stages in the yield formation of late-sown wheat. Other studies have shown that foliar application of high concentrations of TiO_2_ exerts toxic effects on crop yield traits, resulting in a decrease in yield. In this study, under all treatments at different application timings, T2 concentration proved optimal, and the promotional effects decreased when the concentration further increased. This conclusion aligns with the findings of Jaberzadeh [45], jointly verifying that the appropriate application of titanium is key to enhancing wheat yield traits and thereby achieving increased production. In addition, marked differences in yield and quality parameters were recorded across the two-year experimental period, especially in the number of harvested spikes. This may be attributed to limited pre-winter development and reduced cold resistance of late-sown wheat, together with the early onset of low temperatures before winter during the 2025 season and inadequate snow cover, resulting in higher overwintering mortality and a notable decline in population size. This result has also been confirmed by Tian [7]. Although considerable differences in climatic conditions and population structure occurred between years, results from two growing seasons consistently indicated clear enhancement in the photosynthetic physiology, yield, and quality of crops following the application of TiO_2_, highlighting the stability and reliability of the ameliorative effects of TiO_2_.

## 5. Conclusions

Foliar application of TiO_2_ enhanced the photosynthetic pigment content and stress-resistant enzyme activity in the flag leaves of late-sown wheat, thereby sustaining higher photosynthetic physiological capacity in plants. This approach achieved improvements in crop yield and quality. Applying TiO_2_ during the booting stage (S1) increased the grain yield of late-sown wheat by enhancing the differentiation of florets and spikelets, as well as the grain setting rate. Applying TiO_2_ during the flowering stage (S2) enhanced late-sown wheat yield by accelerating grain filling rate, increasing thousand-grain weight, improving grain morphology, and boosting grain storage capacity and plumpness. Notably, TiO_2_ application at the booting stage not only stably increased grain number per spike but also established a better source–sink relationship for grain filling and quality formation at an earlier stage, thereby realizing the effects of TiO_2_ and achieving higher yield and improved grain quality. Additionally, the foliar application of TiO_2_ at a concentration of 501 μmol L^–1^ (T2) had the most favorable promotional effect, simultaneously enhancing grain protein content and related processing quality. Therefore, under local late sowing conditions, spraying 501 μmol L^–1^ (T2) TiO_2_ at the booting stage (S1) appears to be an effective strategy for alleviating the losses caused by late sowing while achieving simultaneous improvements in both high yield and superior quality.

## Figures and Tables

**Table 1:** Physicochemical properties of 10~20 cm soil.

Year	Organic Matter (g kg^–1^)	PH	Total Nitrogen (g kg^–1^)	Total Phosphorus (g kg^–1^)	Total Potassium (g kg^–1^)	Effective Nitrogen (mg kg^–1^)	Effective Phosphorus (mg kg^–1^)	Effective Potassium (mg kg^–1^)
2023–2024	24.46	8.21	0.27	1.16	14.06	46	15.4	143
2024–2025	24.26	8.69	0.32	1.30	7.37	41	38.9	193

**Table 2:** Effects of TiO_2_ spraying time and concentration on yield and yield components of late-sown wheat.

Year	Period	Concentration	Spikes Number (10^4^ hm^–2^)	Grain Number Per Spike	1000-Grain Weight (g)	Yield (kg hm^–2^)
2023–2024	S1	T0	746.39 ± 7.52 ab	38.53 ± 0.31 c	40.11 ± 0.71 a	8298.71 ± 79.06 c
		T1	732.50 ± 6.51 b	39.67 ± 0.50 b	41.03 ± 0.63 a	8450.93 ± 88.02 bc
		T2	746.39 ± 8.83 ab	41.67 ± 0.31 a	41.21 ± 0.21 a	8718.32 ± 96.01 a
		T3	755.00 ± 12.33 a	40.27 ± 0.42 b	41.15 ± 0.67 a	8623.13 ± 190.55 ab
	S2	T0	751.67 ± 5.20 a	38.80 ± 0.53 a	38.97 ± 0.60 c	8214.62 ± 55.27 c
		T1	744.44 ± 3.76 a	36.40 ± 0.60 b	39.71 ± 0.63 bc	8287.43 ± 105.77 bc
		T2	748.33 ± 8.21 a	37.00 ± 0.40 b	41.33 ± 0.47 a	8596.60 ± 81.90 a
		T3	746.39 ± 3.85 a	38.93 ± 0.70 a	40.27 ± 0.57 ab	8412.57 ± 114.97 ab
2024–2025	S1	T0	561.04 ± 8.03 ab	37.35 ± 0.57 c	46.16 ± 0.68 a	6571.16 ± 121.71 c
		T1	569.17 ± 5.40 a	39.00 ± 0.71 b	47.57 ± 0.58 a	6606.28 ± 108.08 c
		T2	554.38 ± 9.21 b	40.95 ± 0.66 a	47.77 ± 0.60 a	7191.89 ± 98.00 a
		T3	556.67 ± 6.45 b	39.30 ± 0.76 b	47.82 ± 0.50 a	6853.00 ± 121.56 b
	S2	T0	558.92 ± 8.95 a	37.30 ± 0.53 a	45.30 ± 0.79 c	6569.91 ± 143.66 c
		T1	566.25 ± 8.28 a	37.15 ± 0.30 a	46.55 ± 0.033 b	6738.18 ± 63.57 b
		T2	558.96 ± 4.23 a	37.45 ± 0.72 a	47.78 ± 0.95 a	6933.91 ± 99.01 a
		T3	555.58 ± 7.80 a	38.00 ± 0.59 a	46.55 ± 0.43 b	6873.01 ± 74.82 ab

Note: S1 and S2 denote TiO_2_ application during the booting stage (S1) and flowering stage (S2), respectively. T0, T1, T2 and T3 indicate the spraying concentrations of TiO_2_ of 0 μmol L^–1^ (T0), 376 μmol L^–1^ (T1), 501 μmol L^–1^ (T2), 626 μmol L^–1^ (T3), respectively. Note: Different letters in the same column indicate significant differences between treatments (*p* < 0.05).

**Table 3:** Effects of TiO_2_ spraying time and concentration on quality of late-sown wheat.

Year	Period	Concentration	Starch Content (%)	Protein Content (%)	Gluten Content (%)	Water Absorption (%)	Sedimentation Value (%)
2023–2024	S1	T0	69.73 ± 0.15 a	12.33 ± 0.06 b	24.63 ± 0.47 b	55.30 ± 0.10 c	41.18 ± 0.05 c
		T1	69.60 ± 0.10 ab	12.57 ± 0.06 a	25.13 ± 0.31 ab	55.70 ± 0.10 b	42.98 ± 0.13 b
		T2	69.50 ± 0.10 b	12.70 ± 0.10 a	25.53 ± 0.25 a	56.13 ± 0.15 a	44.19 ± 0.11 a
		T3	69.53 ± 0.06 ab	12.57 ± 0.15 a	25.20 ± 0.40 ab	55.80 ± 0.10 b	43.10 ± 0.09 b
	S2	T0	69.93 ± 0.15 a	12.13 ± 0.06 b	24.20 ± 0.10 b	55.43 ± 0.12 d	40.71 ± 0.06 d
		T1	69.77 ± 0.06 ab	12.33 ± 0.15 ab	24.63 ± 0.50 ab	56.03 ± 0.15 c	41.08 ± 0.16 c
		T2	69.53 ± 0.15 c	12.50 ± 0.10 a	25.03 ± 0.40 a	56.63 ± 0.12 a	42.57 ± 0.10 a
		T3	69.67 ± 0.06 bc	12.53 ± 0.15 a	24.97 ± 0.31 a	56.30 ± 0.10 b	42.10 ± 0.10 b
2024–2025	S1	T0	68.78 ± 0.10 a	14.75 ± 0.13 c	30.25 ± 0.37 b	55.38 ± 0.10 c	60.30 ± 0.16 d
		T1	68.63 ± 0.10 ab	14.90 ± 0.08 b	30.48 ± 0.34 b	55.70 ± 0.12 b	60.97 ± 0.19 c
		T2	68.35 ± 0.13 c	15.30 ± 0.08 a	31.48 ± 0.53 a	56.05 ± 0.17 a	64.31 ± 0.12 a
		T3	68.58 ± 0.13 b	15.00 ± 0.08 b	30.75 ± 0.19 b	55.95 ± 0.13 a	61.93 ± 0.14 b
	S2	T0	68.88 ± 0.10 a	14.55 ± 0.10 b	29.75 ± 0.21 b	55.70 ± 0.08 b	60.01 ± 0.16 d
		T1	68.75 ± 0.13 a	14.65 ± 0.13 b	29.98 ± 0.10 b	55.90 ± 0.14 ab	60.32 ± 0.16 c
		T2	68.38 ± 0.13 b	15.10 ± 0.08 a	30.98 ± 0.36 a	56.03 ± 0.15 a	62.74 ± 0.10 a
		T3	68.53 ± 0.10 b	15.08 ± 0.13 a	30.90 ± 0.42 a	55.95 ± 0.19 a	62.49 ± 0.14 b

Note: Different letters in the same column indicate significant differences between treatments (*p* < 0.05).

## Data Availability

Due to privacy concerns, the datasets generated during this study are available from the corresponding author upon reasonable request.
